# Risk stratification in patients undergoing interventional left atrial appendage occlusion—Prognostic impact of EuroSCORE II

**DOI:** 10.1002/clc.23338

**Published:** 2020-01-22

**Authors:** Michael Gotzmann, Dinah S. Choudhury, Maximilian Hogeweg, Florian Heringhaus, Andreas Mügge, Andreas Pflaumbaum

**Affiliations:** ^1^ Cardiovascular Center, St. Josef Hospital Bochum, Ruhr‐University Bochum Germany; ^2^ Department of Cardiology Marien Hospital Witten, Ruhr University Bochum Germany

**Keywords:** interventional left atrial appendage occlusion, logistic EuroSCORE II, mid‐term mortality, risk stratification

## Abstract

**Background:**

Interventional closure of the left atrial appendage (LAA) is an alternative option to stroke prophylaxis, particularly in multimorbid patients with a high risk of bleeding under oral anticoagulation. Due to the multiple comorbidities, the prognosis of patients is reduced, and the clinical benefit of the procedure is therefore questionable in the individual patient.

**Hypothesis:**

The present study aims to identify independent preprocedural risk factors to improve risk stratification in these highly selected patients.

**Methods:**

This study consecutively included 128 patients who received an interventional LAA occlusion with Amplatzer device (St Jude Medical, St Paul, Minnesota). The preinterventional risk assessment was performed with the logistic European System for Cardiac Operative Risk Evaluation (EuroSCORE) II. The primary endpoint was all‐cause mortality. Secondary endpoints were thromboembolic events and severe bleeding.

**Results:**

During a follow‐up of 781 ± 498 days the primary endpoint (all‐cause mortality) was reached in 35 patients (27%). The only independent predictor of mid‐term mortality was a logistic EuroSCORE II > 2% (Hazard risk [HR] 4.55, confidence interval [CI] 1.599‐12.966, *P* = .005). In our study, 33 patients (26%) suffered from end‐stage renal disease which was not associated with increased mortality (*P* = .371), increased thromboembolic events (*P* = .475), or severe bleeding (*P* = .613).

**Conclusions:**

In patients undergoing interventional LAA occlusion, preprocedural assessment of logistic EuroSCORE II provide independent prognostic information. This parameter might help to improve risk stratification in these highly selected patients. In contrast, terminal renal failure was not associated with a significantly worse outcome.

## 1 INTRODUCTION

Atrial fibrillation is a common cardiovascular disease with significant morbidity and mortality. There is an increased risk of stroke, which can be significantly reduced by effective anticoagulation.^1^

However, the risk of both stroke and bleeding complications under oral anticoagulation increases significantly with age and the presence of various comorbidities.^2^ In patients with nonvalvular atrial fibrillation, the left atrial appendage (LAA) is clearly the most common location of thrombus formation.^3^ For some years there has been a nonpharmaceutical therapy for prevention of cardioembolic strokes, particularly for high‐risk patients: the interventional occlusion of the LAA.^4^

Previous studies have investigated the rate of immediate procedural success, procedural complications, and efficacy of stroke prophylaxis compared to warfarin.^5^, ^6^, ^7^, ^8^, ^9^ Based on the available studies, the European Society of Cardiology currently give a IIb‐recommendation for interventional LAA occlusion in patients who cannot receive oral anticoagulation due to contraindications.^10^

Of clinical importance is the question of which patients benefit from interventional LAA occlusion. On the one hand, the procedure is associated with a risk of fatal and nonfatal complications. On the other hand, there is a reduced life expectancy due to the comorbidities of patients who are eligible for interventional LAA occlusion. From a clinical point of view, an individual benefit of the therapy may be questionable if life expectancy is too short. To date, there is scarce data on what factors could be prognostically significant to estimate the life expectancy of patients in this particular cohort.^11^, ^12^ The aim of this study is therefore to identify prognostic factors for interventional LAA occlusion with an Amplatzer amulet or an Amplatzer cardiac plug (St Jude Medical, St Paul, Minnesota).

## 2 METHODS

### 2.1 Study design

This study was designed to examine the medium‐term results after interventional LAA occlusion and the prognostic significance of logistic European System for Cardiac Operative Risk Evaluation (EuroSCORE) II. All patients who received an interventional LAA occlusion from November 2012 to December 2017 in the Marien Hospital Witten, Academic Teaching Hospital of the Ruhr‐University Bochum, were included consecutively. The devices used were the Amplatzer Amulet or the Amplatzer cardiac plug (St Jude Medical, St Paul, Minnesota, USA). This study is a retrospective analysis of prospectively gained data. Patients gave informed consent. The study was approved by the local ethics committee of the Ruhr University Bochum (reg. Number 18‐6392).

### 2.2 Inclusion criteria

The inclusion criteria were a history nonvalvular atrial fibrillation, a CHA_2_DS_2_‐VASc (Congestive heart failure, Hypertension, Age ≥ 75 years, Diabetes mellitus, Stroke/TIA, Vascular disease, Age 65‐74 years, Sex category [woman])^13^ score ≥ 2, a contraindication to long‐term oral anticoagulation (previous intracranial hemorrhage or major bleeding, a high risk for bleeding and chronic renal disease requiring dialysis), and in individual cases, the patient's refusal to use anticoagulation. Exclusion criteria were mechanical prosthetic heart valve, life expectancy <1‐year, active endocarditis, and intracardiac thrombus. Indications, contraindications, and anatomical requirements for left atrial appendage occlusion were described previously.^14^, ^15^

### 2.3 Medical history

Cardiovascular diseases and risk factors were diagnosed by medical history, medication, and praeprocedural examinations. New York Heart Association (NYHA) classification was used to assess the symptomatic status of patients. The risk of ischemic stroke was estimated using the CHA_2_DS_2_‐VASc score. The risk of bleeding was estimated using the HAS‐BLED score. The logistic EuroSCORE II was calculated pre‐procedural based on the risk factors of each patient. Measurements of NT‐pro‐BNP and estimated glomerular filtration rate (eGFR) was performed within 48 hours before interventional LAA occlusion.

### 2.4 Device implantation and anticoagulation

**Figure 1:**
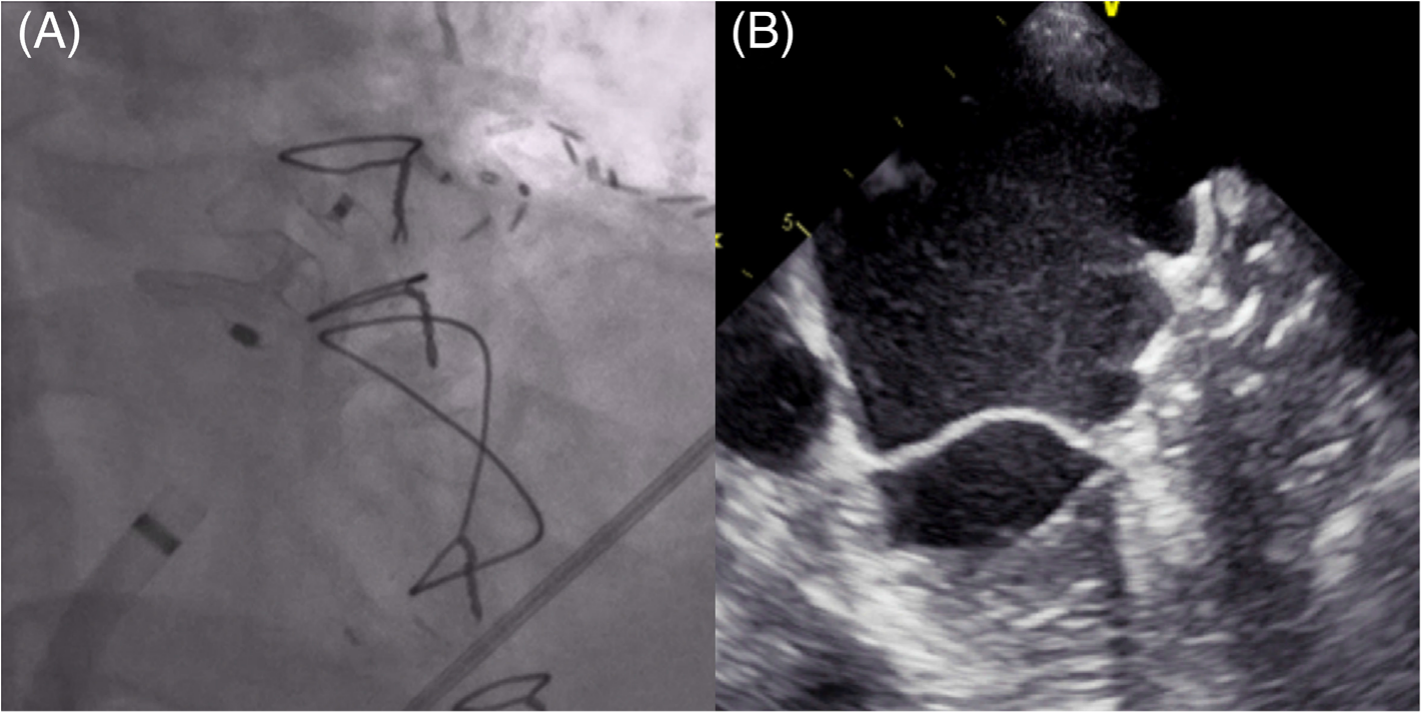
A, Fluoroscopic and B, echocardiographic image of the Amplatzer amulet device

The Amplatzer amulet or the Amplatzer cardiac plug device and the implantation procedure have been described in detail elsewhere.^14^, ^15^ Briefly, the device was implanted under echocardiographic and fluoroscopic guidance via femoral venous access via the transseptal route into the LAA. Accurate device position was confirmed by angiography and echocardiography^14^, ^15^ (Figure 1). After implantation, patients received a dual platelet therapy with aspirin 100 mg/d and clopidogrel 75 mg/d for 1 to 3 months, followed by a monotherapy with aspirin 100 mg/d for at least 6 months or—in the case of corresponding indications—lifelong therapy with aspirin 100 mg/d.

### 2.5 Study end point

The primary study end point was all‐cause mortality, defined as death from any cause after successful LAA occlusion. The secondary end point was defined as (a) hemorrhagic or ischemic stroke, transient ischemic attack (TIA), and other thromboembolic events, and (b) severe bleeding (defined as hemoglobin decrease >2 g/dL, the need for blood transfusion and hospitalization due to bleeding).^16^ Follow‐up information was obtained during routine ambulatory visits but also by telephone contact with the deceased patients' physicians.

### 2.6 Statistics

Numerical values are expressed as mean ± SD. Continuous variables without normal distribution are summarized by the median (first quartile, third quartile). Continuous variables were compared between groups using an unpaired *t* test (for normally distributed variables) or Mann‐Whitney *U* test (for non‐normally distributed variables). χ^2^ analysis was used to compare categorical variables. All variables in Table 2 were evaluated for the primary study end point in a univariate Cox proportional hazard model. All variables with a significant association were entered in a multivariate Cox model to identify independent predictors of outcome. Receiver operating characteristic curves were generated to define cut‐off values for independent predictor. Freedom from all‐cause mortality was analyzed by the Kaplan‐Meier method, and survival curves were compared by the log‐rank test. Results are present as hazard risk (HR). A *P* value less than .05 was considered significant. All probability values reported are two‐sided.

## 3 RESULTS

### 3.1 Procedure and complications

In the period from November 2012 to December 2017, 132 patients underwent the procedure. In one patient, implantation was not possible because the LAA was too small. In another patient the implantation was primarily successful, in the echocardiographic control a dislocation of the device into the aorta ascendens was obvious. The device was recovered via the femoral aorta.

Periprocedural death, stroke or myocardial infarction did not occur in any patients. One patient suffered a tamponade which had to be relieved by pericardial puncture. Procedural details and complications of all patients are listed in Table 1. Two patients with successful device implantation were lost in follow‐up. The remaining 128 patients formed the final study cohort.

**Table 1:** Periprocedural complications in all patients

	Total (n = 132)
Death	0
Myocardial infarction	0
Stroke	0
Device embolization	1
Tamponade	1
Pericardial effusion	3
Inguinal hemorrhage or hematoma	6
Need for transfusion	3
Primary unsuccessful implantation	1

### 3.2 Study cohort

Mean age of 128 study patients (60 women, 68 men) was 76 ± 7.4 years, mean CHA_2_DS_2_‐VASc Score was 4.05 ± 1.3, mean HASBLED score was 4.16 ± 0.66 and mean EuroSCORE II was 2.79% (1.68‐4.19%). Clinical characteristics are provided in Table 2.

**Table 2:** Clinical characteristics of study patients (n = 128)

	Total (n = 128)	Survivors (n = 94)	Nonsurvivors (n = 35)	*P* value
Age (y)	76 ± 7.4	75.9 ± 7.5	76.5 ± 7.1	.645
Women (♀), n (%)	60 (47)	48 (51)	12 (34)	.078
Body mass index (kg/m^2^)	28 ± 5.6	27.7 ± 5.4	28.6 ± 6.1	.425
NYHA class III and IV, n (%)	45 (35)	29 (31)	16 (46)	.154
Left ventricular ejection fraction (%)	51.3 ± 8.5	52 ± 6.8	49.3 ± 11.9	.103
CHA_2_DS_2_‐VASc Score (pts)	4.05 ± 1.3	3.95 ± 1.3	4.34 ± 1.21	.112
HAS‐BLED Score (pts)	4.16 ± 0.66	4.1 ± 0.66	4.31 ± 0.63	.092
Logistic EuroSCORE II (%) (quartile)	2.79 (1.68‐4.19)	2.53 (1.44‐3.7)	3.55 (2.34‐5.61)	.001
Medical history				
Hypertension, n (%)	127 (99)	93 (100)	34 (97)	.271
Diabetes mellitus, n (%)	52 (41)	34 (36)	18 (51)	.157
Coronary artery disease, n (%)	61 (48)	41 (44)	20 (57)	.238
Previous CABG, n (%)	16 (13)	11 (12)	5 (14)	.765
Previous stroke, n (%)	20 (16)	13 (14)	7 (20)	.417
COPD, n (%)	30 (23)	20 (21)	10 (29)	.482
Peripheral artery disease, n (%)	16 (13)	10 (11)	6 (17)	.370
Dialysis, n (%)	33 (26)	22 (23)	11 (31)	.371
Labor				
eGFR (ml/min/1,73 m^2^)	46.8 ± 26.9	50.3 ± 28.4	37.5 ± 20	.018
NT‐pro‐BNP (ng/L) (quartile)	1640 (535‐3475)	1573 (486‐3840)	2175 (1220‐3327)	.267
Procedural details				
Intervention time (min)	63.4 ± 25	62.9 ± 26.5	64.7 ± 20.7	.693
Use of contrast media (mL)	160 ± 96	156 ± 100	169 ± 84	.488
Type of prosthesis	53/75	35/58	18/17	.230

Abbreviations: CABG, coronary artery bypass grafting; COPD, Chronic obstructive pulmonary disease; Dialysis, terminal kidney disease requiring dialysis; eGFR, estimated glomerular filtration rate; NYHA, New York Heart Association; Type of device: Amplatzer cardiac plug vs Amplatzer amulet.

The indications for interventional LAA occlusion were: intracerebral bleeding under anticoagulation (n = 7), prior gastrointestinal bleeding (n = 64), prior other severe bleeding (nasal, pulmonary, vaginal, cutaneous) (n = 24), increased risk of bleeding (n = 28) and refusal of oral anticoagulation (n = 5). In 33 (26%) of 128 study patients terminal renal disease requiring permanent dialysis was present.

After successful implantation, 29 patients received dual antiplatelet therapy with aspirin 100 mg/d and clopidogrel 75 mg/d for 3 months, followed by lifelong aspirin 100 mg/d. The other 99 patients underwent dual antiplatelet therapy with aspirin 100 mg/d and clopidogrel 75 mg/d for one month, followed by 6 months of aspirin 100 mg/d in 38 patients. The remaining 61 patients received clopidogrel for 1 month and aspirin for lifelong. The main reasons for continuing therapy with aspirin was coronary heart disease (n = 61) or peripheral artery disease (n = 16).

In the first 2 to 6 months after successful implantation, 78 patients (61%) underwent a TEE examination in our Hospital. Eight patients revealed a small (≤ 3 mm) leak, a large leak >3 mm was not detected. In one patient a device thrombus was detected 6 months after implantation without any indication for a thromboembolic event. This patient received oral anticoagulation with phenprocoumone for 6 weeks. In the repeat control TEE examination no thrombus was found and the patient received aspirin for life and clopidogrel for another 6 months.

### 3.3 Mortality after LAA occlusion and predictors of all‐cause mortality

During a follow‐up of 781 ± 498 days the primary endpoint (all‐cause mortality) was reached in 35 patients (27%). This corresponds to an annual mortality rate of about 13%. All‐cause mortality in the first year was 13.2% (17 out of 128 patients).

Cardiovascular death occurred in 12 patients: heart failure (n = 5), sudden cardiac death and cardiogenic shock (n = 4), sudden thromboembolic aortic occlusion (n = 1), mesenteric ischemia (n = 1), and postoperative death after heart valve surgery (n = 1). Seventeen patients died due to noncardiovascular causes: pneumonia with respiratory failure (n = 6), sepsis (n = 4), renal failure (n = 2), malignant tumor (n = 3), ileus (n = 1), and severe Parkinson disease (n = 1). In 6 patients the cause of death was unknown.

All variables in Table 2 were evaluated for the primary study end point in a univariate Cox proportional hazard model. On univariate Cox analysis, logistic EuroScore II and eGFR were significantly related to the primary study end point (Table 3). All variables with a significant association were entered in a multivariate Cox model to identify independent predictors of outcome. Stepwise multivariable analysis identified only logistic EuroScore II as an independent predictor of all‐cause mortality (Table 3).

**Table 3:** Univariate and multivariate analysis

	Hazard ratio	CI	*P* value
Univariate analysis			
EuroSCORE II (%)	1.15	1.044‐1.272	.005
eGFR	0.98	0.968‐0.997	.019
Multivariate analysis			
EuroSCORE II > 2%	4.55	1.599–12.966	.005

Using receiver operating characteristic analysis, cut‐off values for separating study patients was logistic EuroScore II > 2% (area under curve [AUC]: 0.68; CI 0.587‐0.778, *P* < .001). Patients with a logistic EuroScore II > 2% had a hazard ratio of 4.55 (CI 1599‐12 966, *P* = .005). Kaplan‐Meier curves were generated for all‐cause mortality (Figure 1). Patients with a logistic EuroScore II ≤2% had a total mortality of 4.5% compared to 18% in patients with a logistic EuroScore II > 2% (*P* < .002).

To analyze the significance of each parameter of the logistic EuroSCORE II, patients with a logistic EuroSCORE II > 2% were compared with patients with a logistic EuroSCORE ≤2%. Patients with a logistic EuroSCORE II > 2% were, for example, older, had more frequent diabetes mellitus, coronary heart disease, poorer left ventricular ejection fraction and poorer renal function (TABLE S1).

### 3.4 Secondary endpoints—stroke, thromboembolic events, and bleeding

During the study period of about 2.14 years, 5 (3.9%) strokes/TIA occurred. One stroke occurred after 20 days and was associated with death, so that a procedural or device‐associated complication can be assumed. The other 4 strokes/TIA occurred more than 1 year after occluder implantation. The mean CHA_2_DS_2_‐VASc score of the patients was 4.05 with a predicted risk of stroke of about 4% per year.^13^ Thus, the patients in our study had a reduction of expected stroke rate by about 55%.

A total of two patients (1.5%) suffered thromboembolic events (one sudden thromboembolic aortic occlusion and one mesenteric ischemia). The annual rate of strokes/TIA and other thromboembolic events was 2.5%. In the group of patients requiring dialysis (n = 33, 26%) one thromboembolic event occurred. There was no significant difference in the number of thromboembolic events between patients with end‐stage renal disease compared to patients without end‐stage renal disease (*P* = .475).

During the entire study period, nine patients (7%) suffered from severe bleeding, which corresponds to an annual rate of 3.3%. The mean HAS BLED score was 4.16 with a predicted rate for severe bleeding of about 8.9% per year.^16^ Compared to the predicted bleeding rate, the observed bleeding rate in the study was 63% lower. In the group of patients requiring dialysis (n = 33, 26%) three severe bleeding occurred. There was also no significant difference in the number of severe bleedings between patients with terminal renal failure compared to patients without end‐stage renal disease (*P* = .613).

## 4 DISCUSSION

The present study examined the mid‐term results after interventional LAA occlusion with an Amplatzer cardiac plug and an Amplatzer amulet. The main finding of the study was that the logistic EuroScore II was the only independent risk factor for mid‐term all‐cause mortality. Patients with a logistic EuroScore II > 2% had a significantly higher probability of all‐cause mortality compared to patients with a logistic EuroScore II ≤2% (hazard ratio of 4.55 [CI 1.599‐12.966]) (Figure 1). The logistic EuroScore II thus may allow a simple estimation of the prognosis of patients undergoing interventional LAA closure. Risk stratification could improve the selection of patients who, due to their limited prognosis, have probably little or no benefit to expect from an interventional LAA occlusion.

### 4.1 Outcome of LAA occlusion

In our study we observed a procedural success rate of 98%. In one patient the implantation of the device was not possible with a too small LAA, in another patient dislocation occurred in the first hours after implantation (Table 1). The rate of procedural success is comparable to the results of other recent studies with the Amplatzer devices.^8^, ^9^, ^17^, ^18^, ^19^ The devices we used were the Amplatzer cardiac plug and the Amplatzer amulet. In earlier studies it could be demonstrated that both prostheses had no significant difference in the results.^20^, ^21^ Also, in our study, there was no difference in all‐cause mortality regardless of the device selection (Table 2).

In the present study, the mean CHA_2_DS_2_‐VASc score of patients was 4.05 points with an expected stroke rate of approximately 4% per year. The actual annual stroke rate after interventional LAA occlusion was 1.8%. However, two thromboembolic events occurred, so that the annual rate for stroke, TIA and thromboembolic events was approximately 2.5%. This result confirms the results of previous studies with Amplatzer devices.^8^, ^9^, ^17^, ^18^

The total mortality in our study was 13% per year. This relatively high mortality is due to the relatively high age of patients with multiple comorbidities (Table 2). In particular, the proportion of patients with end‐stage of kidney disease requiring permanent dialysis was larger in our collective than in other studies on LAA occlusion. The noncardiac death was responsible for about half of the deaths, the cardiac death for about one third, while the remaining deaths remained unclear. In our opinion, the relatively high mortality of the study patients, especially in the mid‐term, justifies the search for suitable factors for the risk stratification of patients undergoing interventional LAA occlusion. In a recent publication, Koskinas et al identified the need for device repositioning and a left ventricular ejection fraction <30% as risk factors for procedure‐ and device‐related major adverse events.^12^ However, the study only investigated complications in the first 7 days and did not investigate long‐term mortality.

In the study by Regueiro et al. One‐hundred and one patients with an average of 4.1 years were examined who underwent an interventional LAA occlusion.^11^ This study identified older age, male sex, low ejection fraction, and chronic kidney disease as predictive factors of late mortality. In contrast, in our study only eGFR and EuroSCORE II were associated with higher all‐cause mortality (Table 3).

### 4.2 Patients with kidney disease undergoing interventional LAA occlusion

In our study, a lower eGFR was associated with a significantly higher all‐cause mortality (Tables 2 and 3). Our result is thus consistent with the result of the aforementioned study by Regueiro et al.^11^ However, eGFR was not an independent risk factor (Table 3).

In contrast, our study suggests that end‐stage renal disease, which requires permanent dialysis, is not a significant risk factor for postinterventional outcome. In our study, 33 patients (26%) had end‐stage renal disease (Table 2). There was no difference in all‐cause mortality between patients with and without end‐stage renal disease (Table 2). In addition, there was no difference between the two groups in the frequency of thromboembolic events or severe bleeding. To date, very little data is available on the treatment of patients with end‐stage renal disease. Kefer et al were able to demonstrate that patients with kidney disease can be safely and effectively treated with the interventional LAA occluder.^22^ In particular, patients with severe GFR impairment may benefit from the reduction of thromboembolic and bleeding conditions. However, in this study, only 14 patients had end‐stage renal disease.^22^ In the study by Genovesi et al, a total of 50 patients with terminal renal failure who underwent interventional LAA occlusion were examined. The preliminary results showed a good outcome after 30 days without death, stroke or bleeding.^23^

Our study can thus support the results of the above studies that the interventional LAA occlusion could be a suitable procedure for the prevention of strokes and bleeding in patients with end‐stage renal disease. In addition, we were able to demonstrate in our study the favorable medium‐term results in this particular patient group.

### 4.3 Logistic EuroScore II

The well validated logistic EuroScore II was developed for the estimation of short‐term mortality after cardiac surgery and includes a variety of clinical parameters which are weighted differently.^24^ In recent years, the logistic EuroSCORE II has played an important role in the question of whether a patient should undergo cardiac surgery or interventional heart valve implantation.^25^ Nearly all current studies used the logistic EuroSCORE II to illustrate the risk profile of patients. Furthermore, the logistic EuroSCORE has prognostic implications in patients undergoing TAVI or MitraClip.^26^, ^27^ The EuroSCORE II is suitable for numerous procedures and therefore offers more flexibility than other scores.^28^

**Figure 2:**
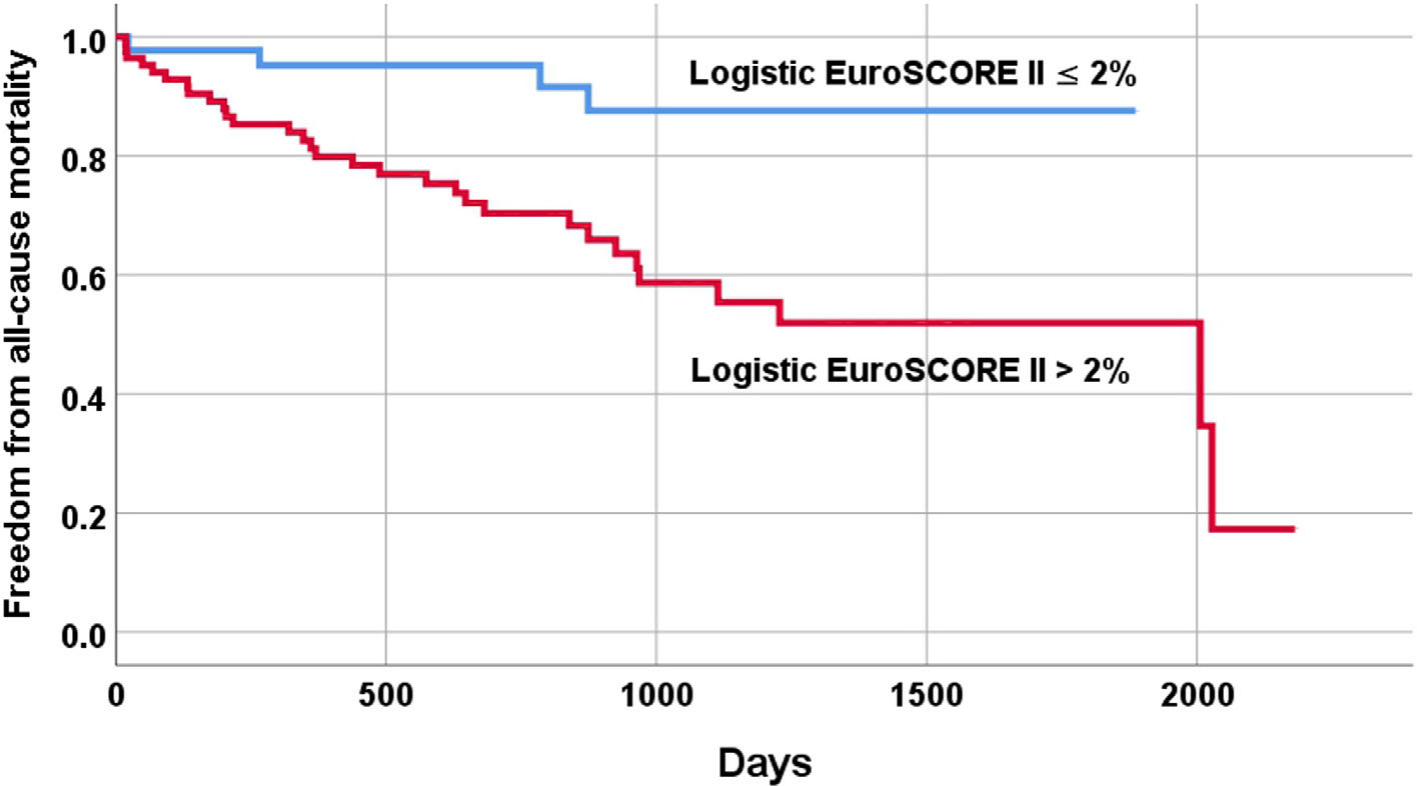
Risk model based on independent predictor of all‐cause mortality: Kaplan‐Meier estimates of freedom from all‐cause mortality

Our study is the first to investigate the prognostic significance of the logistic EuroSCORE II for interventional LAA occlusion. In our study cohort an increased logistic EuroSCORE II was associated with a higher mid‐term mortality and it was the only independent risk factor for all‐cause mortality (Table 3). A logistic EuroSCORE II > 2% was able to differentiate between patient groups with low and high medium‐term mortality (Figure 2). It should be noted that only the logistic EuroSCORE II had a prognostic significance in our patients and not the individual factors such as age or left ventricular ejection fraction. This is probably due to the fact that the number of study patients was relatively small, so that only a risk score that considered many factors together showed a significant difference in the mid‐term mortality (TABLE S1).

Risk stratification of patients is a clinical challenge to identify patients who are unlikely to benefit from interventional LAA occlusion due to their low life expectancy. The EuroSCORE II calculates an operational risk from 17 easily identifiable factors. These 17 factors can be determined by routine anamnesis, laboratory tests and echocardiography before a planned closure of the LAA. A calculated high EuroSCORE II should possibly lead to a reconsideration of the indication for LAA closure, as patients with significantly reduced life expectancy may not benefit clinically from LAA closure. This is particularly true for patients with a relatively low CHA2DS2‐VASc score^2^, ^3^ and a high EuroSCORE II. In these patients, the annual risk of stroke could be lower than the annual risk of dying from nonthrombotic causes. Our results might be the first step for developing a specific risk score in patients undergoing interventional LAA occlusion.

### 4.4 Limitations

The present study only investigated patients who underwent interventional LAA occlusion with an Amplatzer amulet or an Amplatzer cardiac plug. Therefore, the results may not apply to patients who underwent LAA occlusion with another devices.

In our study we could not provide the frequency of leaks and device‐related thrombus. This is due to the circumstance that the postinterventional TEE follow‐up examinations were only partially performed in our clinic. However, this study was designed to investigate the overall mortality of the patients.

The main limitation is the relatively small sample size and the and the retrospective character of the study. However, follow‐up succeeded in the vast majority of patients; less than 2% of the patients were lost in the follow‐up. In addition, it should be noted that this study is the first to perform risk stratification in patients undergoing interventional LAA occlusion.

## 5 CONCLUSIONS

The present study confirmed the positive effects of interventional LAA occlusion on the reduction of stroke and severe bleeding. Remarkably, in the group of patients with end‐stage renal disease there was neither an increased mortality nor an increased rate of thromboembolic events or bleeding. In addition, the study highlights the importance of risk stratification in patients, as only patients with longer life expectancy can expect the positive effects of interventional LAA occlusion. Our study suggests that the logistic EuroSCORE II could be an important factor for the development of an LAA closure risk score.

## CONFLICT OF INTEREST

Dr. Gotzmann is a consultant for and received funding from Abbott, the other authors report no conflict of interest.

## Supporting information


**TABLE S1** Clinical characteristics of study patients (EuroSCORE > 2% vs ≤ 2%) (n = 128)
