# Supplementary material for: Risk stratification in patients undergoing interventional left atrial appendage occlusion—Prognostic impact of EuroSCORE II
Source: Clin Cardiol. 2020 Jan 22;43(5):508–15. doi: 10.1002/clc.23338 (PMC7244292; doi:10.1002/clc.23338)
Supplement: Supplementary file 1 — TABLE S1 Clinical characteristics of study patients (EuroSCORE > 2% vs ≤ 2%) (n = 128) [file CLC-43-508-s001.docx]

**Table supplement** Clinical characteristics of study patients (EuroSCORE > 2% vs. ≤ 2%) (n = 128)

|  | Total  (n = 128) | EuroSCORE > 2%  (n = 84) | EuroSCORE ≤ 2%  (n = 44) | p value |
| --- | --- | --- | --- | --- |
| Age (years) | 76 ± 7.4 | 72.3 ± 7.9 | 78 ± 6.3 | <0.001 |
| Women (♀), n (%) | 60 (47) | 39 (46) | 21 (48) | 0.889 |
| Body mass index (kg/m²) | 28 ± 5.6 | 28.2 ± 5.9 | 27.4 ± 5.1 | 0.423 |
| NYHA class III & IV, n (%) | 45 (35) | 39 (46) | 6 (14) | <0.001 |
| Left ventricular ejection fraction (%) | 51.3 ± 8.5 | 49.8 ± 9.4 | 54.1 ± 5.4 | 0.007 |
| CHA_2_DS_2_-VASc Score (pts) | 4.05 ± 1.3 | 4.51 ± 1.2 | 3.18 ± 0.97 | <0.001 |
| HAS-BLED Score (pts) | 4.16 ± 0.66 | 4.33 ± 0.59 | 3.82 ± 0.66 | <0.001 |
| Medical history |  |  |  |  |
| Hypertension, n (%) | 127 (99) | 83 (99) | 44 (100) | 0.467 |
| Diabetes mellitus, n (%) | 52 (41) | 47 (56) | 5 (51) | <0.001 |
| Coronary artery disease, n (%) | 61 (48) | 50 (60) | 11 (25) | <0.001 |
| Previous CABG, n (%) | 16 (13) | 15 (18) | 1 (2) | 0.011 |
| Previous stroke, n (%) | 20 (16) | 16 (19) | 4 (9) | 0.141 |
| COPD, n (%) | 30 (23) | 25 (30) | 5 (11) | 0.020 |
| Peripheral artery disease, n (%) | 16 (13) | 14 (17) | 2 (5) | 0.049 |
| Dialysis, n (%) | 33 (26) | 27 (32) | 6 (14) | 0.023 |
| Labor |  |  |  |  |
| eGFR (ml/min/1,73 m²) | 46.8 ± 26.9 | 38.8 ± 22.1 | 62.1 ± 28.9 | <0.001 |
| NT-pro-BNP (ng/l) (Quartile) | 1640 (535 – 3475) | 2089 (1183 – 5984) | 745 (403 – 2210) | 0.045 |
| Procedural details |  |  |  |  |
| \| Intervention time (min) \| \| --- \| | 63.4 ± 25 | 64.9 ± 25.4 | 60.6 ± 24.3 | 0.365 |
| Use of contrast media (ml) | 160 ± 96 | 159 ± 95 | 161 ± 99 | 0.887 |
| Type of prosthesis | 53/75 | 34/50 | 19/25 | 0.770 |

NYHA, New York Heart Association; CABG, coronary artery bypass grafting; COPD, Chronic obstructive pulmonary disease; Dialysis, terminal kidney disease requiring dialysis; eGFR, estimated glomerular filtration rate; Type of device: Amplatzer cardiac plug vs. Amplatzer amulet
